# Paradoxical respiratory failure due to cryptococcal pneumonia after amphotericin B treatment for HIV-associated cryptococcal meningitis

**DOI:** 10.1016/j.mmcr.2017.02.004

**Published:** 2018-01-17

**Authors:** James E. Scriven, Francois CJ Botha, Charlotte Schutz, David G. Lalloo, Helen Wainwright, Graeme Meintjes

**Affiliations:** a Clinical Infectious Diseases Research Initiative, Institute of Infectious Disease and Molecular Medicine, University of Cape Town, Observatory, 7925, South Africa; b Liverpool School of Tropical Medicine, L3 5QA, UK; c Department of Pathology, University of Cape Town and Groote Schuur Hospital, Observatory, 7925, South Africa; d Department of Medicine, University of Cape Town and Groote Schuur Hospital, Observatory, 7925, South Africa

**Keywords:** Cryptococcus neoformans, Respiratory failure, Cryptococcal pneumonia, Amphotericin B deoxycholate, Paradoxical deterioration

## Abstract

We present a 27-year-old lady with HIV-1 infection who died due to rapidly worsening respiratory failure one day after commencing amphotericin B deoxycholate therapy for cryptococcal meningitis. Chest x-ray appearances were consistent with pneumocystis pneumonia but post mortem examination showed evidence of severe necrotizing cryptococcal pneumonia. Cryptococcal pneumonia is an underrecognized condition and should be considered in the differential of patients with HIV-1 infection and low CD4 count who develop respiratory symptoms.

## 1 Introduction

Cryptococcosis is an invasive mycosis, caused by the environmental yeast *Cryptococcus neoformans*. Infection occurs via the inhalational route and although exposure is common, invasive disease is usually restricted to individuals with impaired cell-mediated immunity [1]. Due to the ongoing HIV epidemic it remains a significant cause of morbidity and mortality in sub-Saharan Africa [2], [3]. The most commonly recognized and severe presentation is cryptococcal meningitis but other organ systems can also be affected including the lungs, skin, and the eye [4]. Here we present a fatal case of HIV-associated cryptococcal meningitis complicated by rapidly worsening respiratory failure after commencing amphotericin B deoxycholate anti-fungal therapy. Post mortem examination of the lungs revealed the underlying cause to be necrotizing cryptococcal pneumonia. The patient had been enrolled into a cohort study in Cape Town, South Africa and had provided written informed consent. The University of Cape Town Human Research Ethics Committee granted ethical approval for the study (408/2010 and 371/2013).

## 2 Case

A 27-year old woman with established HIV-1 infection presented to hospital with a one-month history of headache, neck stiffness, vomiting, blurred vision and cough (Day 0). She had been started on anti-retroviral therapy (stavudine, lamivudine and efavirenz) five years previously but three months prior to admission (Day −90) had been changed to zidovudine, lamivudine, and lopinavir/ritonavir by her local clinic after her CD4 count was measured at 24 cells/μL. When she arrived at hospital she was apyrexial, her heart rate was 118 beats per minute, her blood pressure was 104/64 mmHg, respiratory rate was 20 breaths per minute and oxygen saturations 95% on room air. There was no impairment of consciousness, she had mild meningism but no focal neurological abnormalities and normal fundoscopy; her chest was clear to auscultation. A lumbar puncture was performed: opening pressure was not raised (11 cmH_2_0), cerebrospinal fluid (CSF) leukocyte count was normal (3 cells/μL, all lymphocytes), CSF protein was 0.56 g/L and glucose 2.7 mmol/L (serum glucose 5.1 mmol/L). India ink examination revealed the presence of encapsulated yeasts and CSF cryptococcal antigen test was positive. Other blood tests revealed a macrocytic anaemia (Hb 8.1 g/L, MCV 108 fL), reduced albumin (19 g/L), elevated C-reactive protein (95.4 mg/L), but normal renal and hepatic function.

**Fig. 1:**
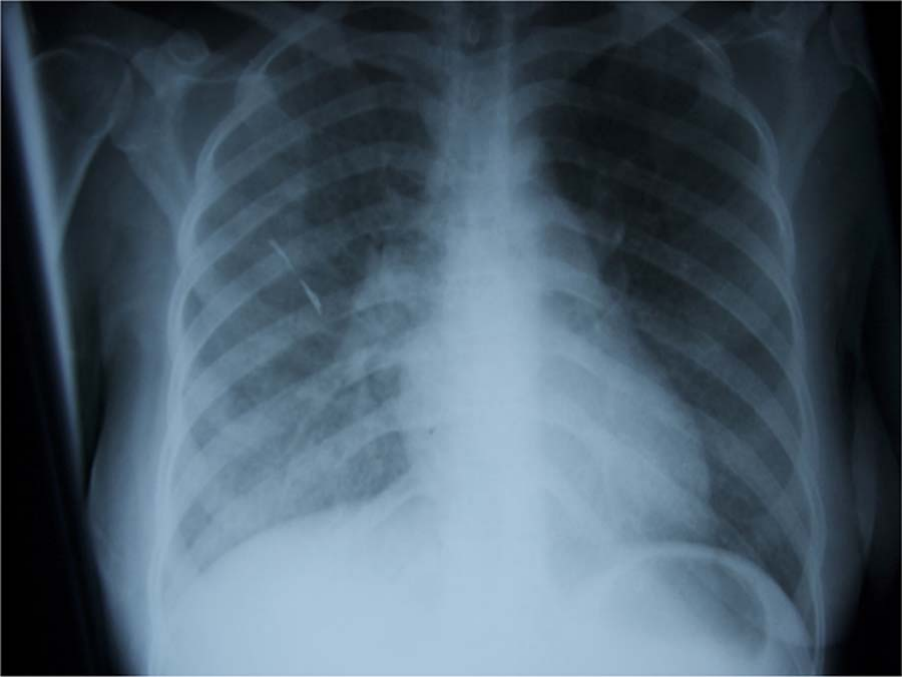
Chest X-ray on Day+1, diffuse interstitial shadowing noted throughout both lung fields, greater on the right.

She was diagnosed with cryptococcal meningitis (Day 0) and started on intravenous amphotericin B deoxycholate 1 mg/kg daily with oral fluconazole 800 mg daily as per local guidance [5]. Anti-retroviral medications were continued. The following day (Day+1) she deteriorated markedly with worsening breathlessness. On examination her heart rate was 150 beats per minute, respiratory rate was 35 breaths per minute and oxygen saturation was 80% breathing room air. Her chest was clear to auscultation but a chest x-ray showed bilateral diffuse ground glass changes more extensive on the right side [Fig. 1].

A presumptive diagnosis of *Pneumocystis jirovecii* pneumonia was made and she was treated empirically with high dose co-trimoxazole (1920 mg three times daily) and prednisone 40 mg twice daily along with high flow oxygen (Day+1). Although her condition initially stabilized she deteriorated the following day with worsening respiratory failure and died (Day+2). Her family provided written informed consent for post mortem examination. Subsequent results revealed a CD4 count of 45 cells/μL and HIV viral load 296 copies/mL (Day+3); *Cryptococcus neoformans* was isolated from both cerebrospinal fluid and blood (Day+3).

**Fig. 2:**
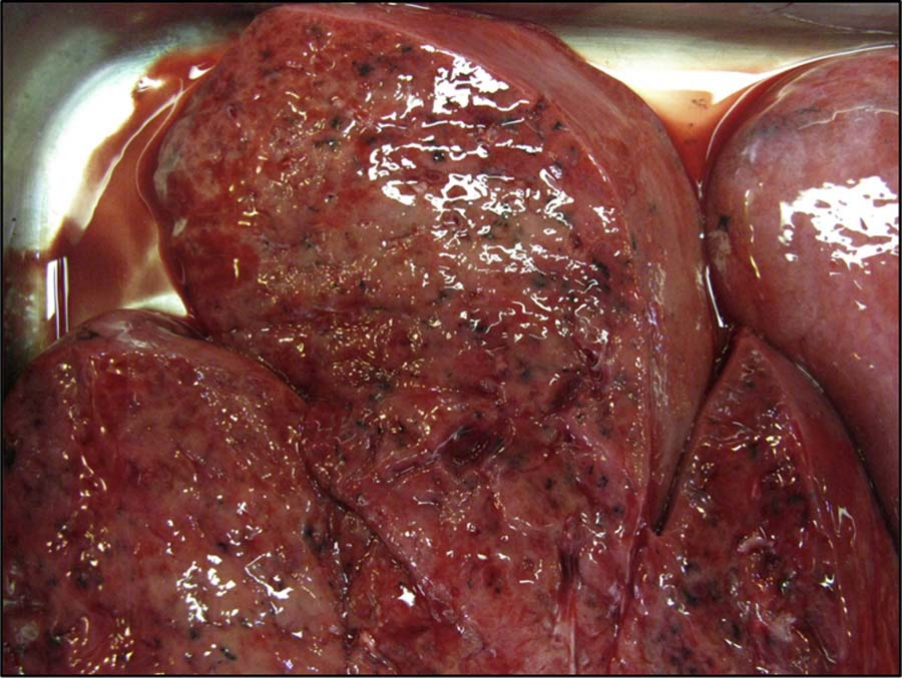
Cut surface of the fresh lung demonstrating poorly demarcated areas of consolidation with thick mucoid secretions. (For interpretation of the references to color in this figure legend, the reader is referred to the web version of this article.)

At post mortem (Day+7), both lungs showed elevated firm, grey-red, poorly demarcated areas of consolidation. There was no cavity formation or caseous necrosis and the cut surface showed accumulations of thick mucoid secretions [Fig. 2].

**Fig. 3:**
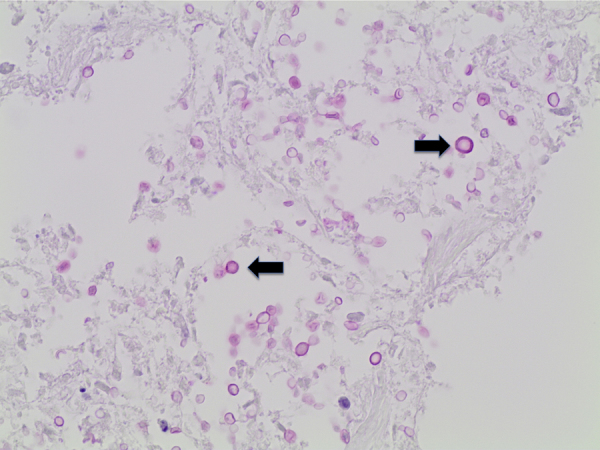
Lung tissue (mucicarmine stain, x40), numerous encapsulated yeasts are seen (marked by black arrow).

**Fig. 4:**
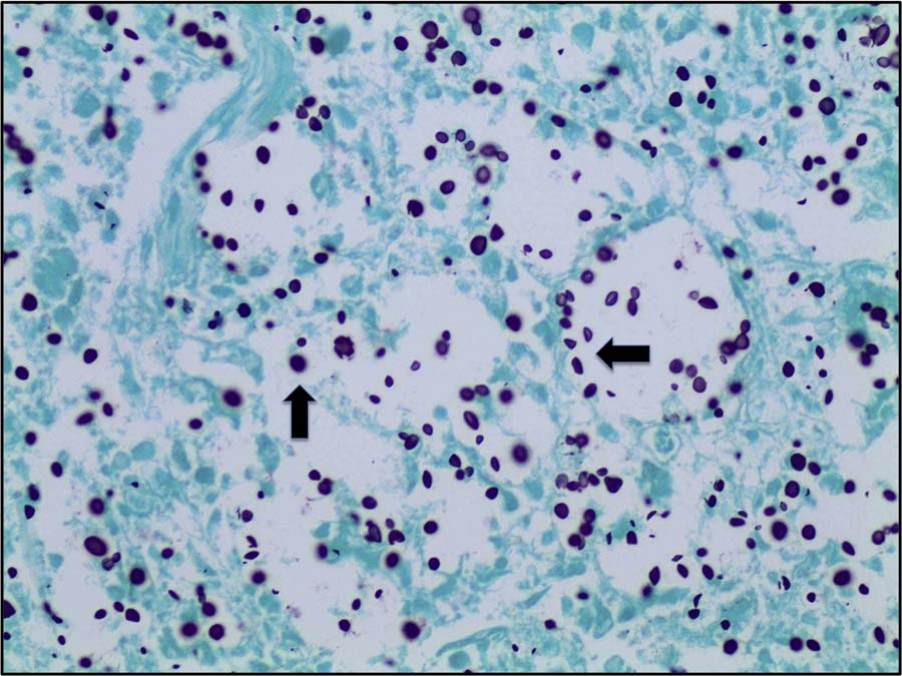
Lung tissue (Grocott's stain, x40), numerous cryptococci are seen throughout the section, stained dark purple (marked with black arrow).

**Fig. 5:**
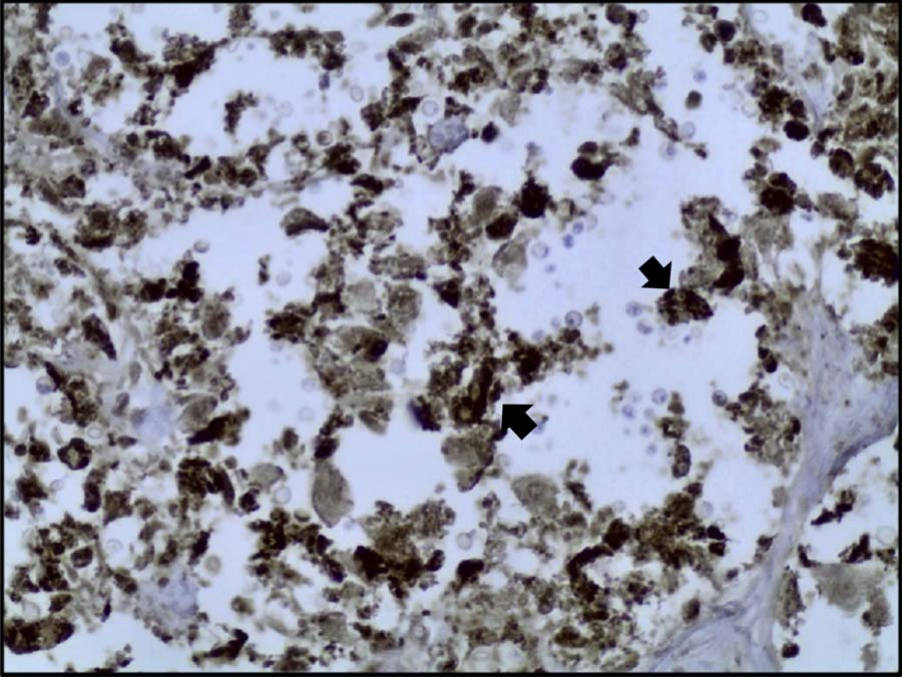
Lung tissue with CD68 immunoperoxidase stain highlighting alveolar macrophages with dark brown intracytoplasmic staining (see arrows; x40).

Histological examination showed diffuse expansion of almost all airspaces by abundant mucoid colonies of encapsulated yeasts, highlighted by Periodic acid–Schiff (PAS), Southgate's mucicarmine [Fig. 3] and Grocott's Gomori's Methenamine Silver stains [Fig. 4]. The alveolar septae appeared slightly thickened by a mild infiltrate of lymphocytes and macrophages [Fig. 5]. No histological evidence for pneumocystis pneumonia was found; the cause of death was recorded as cryptococcal pneumonia.

## 3 Discussion

Although meningoencephalitis is the most widely recognized manifestation of cryptococcosis, pulmonary involvement is well described [6]. The clinical presentation of cryptococcosis is highly dependent on the host immune response. In an immune competent host the infection may be asymptomatic and localized solely to the lung while in the context of profound immune suppression pulmonary involvement can be extensive and part of a wider, disseminated, infection. This is demonstrated by post-mortem studies of persons who had died from HIV-associated cryptococcal meningitis, which have shown histopathological evidence of pulmonary cryptococcosis in 38–52% of cases [7], [8]. Typical symptoms of pulmonary cryptococcosis include cough, fever, chest pain, weight loss, dyspnoea, and haemoptysis while chest x-ray changes include pulmonary nodules, masses, lymphadenopathy, lobar consolidation, reticulonodular infiltrates and pleural effusions [6].

Given the non-specific clinical features, it is highly likely that pulmonary cryptococcosis is under-recognized by clinicians. Among patients with HIV-1 infection it may be misdiagnosed as tuberculosis, bacterial pneumonia or *Pneumocystis jirovecii* pneumonia [9]. This was emphasized by a post mortem study of the lungs of South African miners in which histological evidence of pulmonary cryptococcosis was noted in 589 of 8421 miners (7%). While a respiratory cause of death was recorded for 263 of these miners (44.7%), only 7 (2.7%) were diagnosed with cryptococcal pneumonia prior to their death. Instead miners were erroneously diagnosed as having pulmonary tuberculosis (66.2%) or bacterial pneumonia (24%) [8].

Respiratory failure has been previously described in pulmonary cryptococcosis with retrospective studies from the USA prior to the development of anti-retroviral therapy reporting it in up to 9% of patients [9]. In our recent prospective cohort study in Cape Town, acute respiratory failure developed in 3% (2 of 60) individuals treated for HIV-associated cryptococcal meningitis [10]. In both cases respiratory disease was not evidence clinically at presentation to hospital but developed rapidly after commencing treatment with amphotericin B deoxycholate and fluconazole. This time course suggests that the respiratory deterioration may have been provoked by starting anti-fungal therapy. Possible mechanisms for this include the release of antigens following fungal killing or amphotericin B-mediated release of pro-inflammatory cytokines [11]. Given the switch in anti-retroviral therapy 3 months prior to presentation, it is also possible that unmasking cryptococcal immune reconstitution inflammatory syndrome may have played an additional role [12].

Given these findings, there should be a greater awareness of the non-neurological manifestations of cryptococcosis, especially in patients with disseminated disease in the context of immune deficiency. Further work is also required to determine the incidence of acute respiratory deterioration following initiation of amphotericin B therapy and the pathophysiology behind this.

## Conflict of interest

None of the authors have any conflicts of interests to declare.

## References

[1] Davis J., Zheng W.Y., Glatman-Freedman A., Ng J.A.N., Pagcatipunan M.R., Lessin H. (2007). Serologic evidence for regional differences in pediatric cryptococcal infection. Pedia. Infect. Dis. J..

[2] Park B.J., Wannemuehler K.A., Marston B.J., Govender N., Pappas P.G., Chiller T.M. (2009). Estimation of the current global burden of cryptococcal meningitis among persons living with HIV/AIDS. AIDS.

[3] Jarvis J.N., Meintjes G., Williams A., Brown Y., Crede T., Harrison T.S. (2010). Adult meningitis in a setting of high HIV and TB prevalence: findings from 4961 suspected cases. BMC Infect. Dis..

[4] Dromer F., Mathoulin S., Dupont B., Laporte A. (1996). Epidemiology of cryptococcosis in France: a 9-year survey (1985–1993). Clin. Infect. Dis..

[5] Govender N., Meintjes G., Bicanic T., Dawood H., Harrison T.S., Jarvis J. (2013). Guideline for the prevention, diagnosis and management of cryptococcal meningitis among HIV-infected persons: 2013 update. South Afr. J. HIV Med..

[6] Shirley R.M., Baddley J.W. (2009). Cryptococcal lung disease. Curr. Opin. Pulm. Med..

[7] Klock C., Cerski M., Goldani L.Z. (2009). Histopathological aspects of neurocryptococcosis in HIV-infected patients: autopsy report of 45 patients. Int. J. Surg. Pathol..

[8] Wong M.L., Back P., Candy G., Nelson G., Murray J. (2007). Cryptococcal pneumonia in African miners at autopsy. Int. J. Tube. Lung Dis..

[9] Visnegarwala F., Graviss E.A., Lacke C.E., Dural A.T., Johnson P.C., Atmar R.L. (1998). Acute respiratory failure associated with cryptococcosis in patients with AIDS: analysis of predictive factors. Clin. Infect. Dis..

[10] Scriven J.E., Graham L.M., Schutz C., Scriba T.J., Wilkinson K.A., Wilkinson R.J. (2016). A Glucuronoxylomannan-associated immune signature, characterized by monocyte deactivation and an increased interleukin 10 level, Is a predictor of death in cryptococcal meningitis. J. Infect. Dis..

[11] Mesa-Arango A.C., Scorzoni L., Zaragoza O. (2012). It only takes one to do many jobs: amphotericin B as antifungal and immunomodulatory drug. Front Microbiol..

[12] Haddow L.J., Colebunders R., Meintjes G., Lawn S.D., Elliott J.H., Manabe Y.C. (2010). Cryptococcal immune reconstitution inflammatory syndrome in HIV-1-infected individuals: proposed clinical case definitions. Lancet Infect. Dis..

